# Approaches to Reduce Toxicity in Pediatric Brain Tumors

**DOI:** 10.3390/curroncol32050281

**Published:** 2025-05-15

**Authors:** Hallie Coltin, Christina Coleman, Chantel Cacciotti

**Affiliations:** 1 Division of Hematology/Oncology, Department of Pediatrics, CHU Sainte-Justine, Université de Montréal, Montréal, QC H3C 3A7, Canada; hallie.coltin.med@ssss.gouv.qc.ca; 2 Azrieli Research Center, CHU Sainte-Justine, Montréal, QC H3T 1C5, Canada; 3 Division of Hematology/Oncology, Montreal Children’s Hospital, McGill University Health Centre, Montreal, QC H4A 3J1, Canada; christina.coleman.med@ssss.gouv.qc.ca; 4 Division of Hematology/Oncology, Department of Pediatrics, London Health Sciences Centre & Western University, London, ON N6A 5W9, Canada

**Keywords:** pediatric brain tumors, late effects, ototoxicity, neurocognitive, vascular, radiation necrosis, de-escalation, fertility, surveillance

## Abstract

Pediatric central nervous system (CNS) tumor survivors are highly susceptible to long-term toxicity due to tumor location and also the treatment received. Advancements in treatment techniques, risk-adapted approaches to therapy with adjustments to treatment regimens—including de-escalation when feasible—along with the addition of supportive therapy and surveillance in these survivors, serve to minimize and manage late effects of therapy.

## 1. Introduction

**Figure 1:**
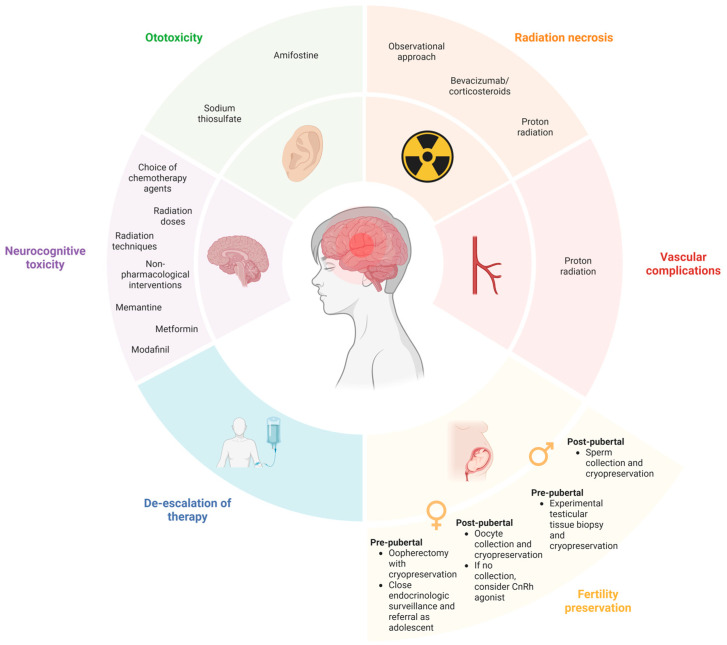
Approaches to reduce and manage toxicity in pediatric CNS tumors. Created in BioRender. Coleman, C. (2025), https://app.biorender.com/illustrations/6821fce9f3f9838a242e30d7?slideId=3b25a4e6-5e03-4921-8c76-57af3cd2b260, accessed on 5 April 2025.

Central nervous system (CNS) tumors represent the most common solid tumors of childhood [1,2,3]. Importantly, this disease group is the leading cause of cancer-related morbidity and mortality among children [3]. Survivors of pediatric CNS tumors are highly susceptible to long-term toxicity resulting from tumor location and receiving intensive treatment [4,5,6]. Recent biological advances in tumor classification empowered the pediatric neuro-oncology community to prioritize therapy de-escalation when safe and pursue feasible toxicity mitigation strategies and targeted long-term surveillance for these survivors. In this review, we discuss the most frequent challenges faced by pediatric CNS tumor survivors as well as recent advances to minimize long-term treatment-related sequelae (Figure 1).

## 2. Ototoxicity

Ototoxicity, defined as hearing loss, tinnitus, and/or vertigo [7], is a frequent and permanent late effect among children diagnosed with CNS tumors [6,8,9,10]. The development of ototoxicity is strongly associated with decreased quality of life, social isolation, adverse mental health outcomes, and educational challenges [11,12,13,14,15,16]. The causes of ototoxicity are multifactorial and include structural damage during neurosurgical interventions, hydrocephalus, and cochlear damage from radiation therapy and platinum-based chemotherapy [6,12,17,18,19,20,21]. Genetic susceptibility may also impact the risk of developing ototoxicity from platinum agents [22]. Given the significant morbidity associated with ototoxicity and the frequent use of platinum-based agents (predominantly cisplatin and carboplatin) as chemotherapy backbones in pediatric neuro-oncology, recent efforts have focused on ototoxicity prevention. The most commonly investigated systemically administered agents have been amifostine and sodium thiosulfate (Figure 1) [23].

Amifostine, an organic thiophosphate compound, is dephosphorylated by alkaline phosphatase to its active metabolite, which binds to cisplatin metabolites and scavenges free radicals [23]. While amifostine was included as an otoprotective agent in the recent medulloblastoma SJMB12 trial (NCT01878617), a recent systematic review strongly recommends against its use due to a lack of efficacy in preventing ototoxicity (Table 1) [23,24].

On the contrary, sodium thiosulfate (STS) is commercially available in the United States and Europe for the prevention of cisplatin-induced hearing loss for children, adolescents, and young adults with non-metastatic solid tumors [25,26,27]. STS likely acts to reduce cisplatin-induced ototoxicity by promoting the formation of inactive cisplatin compounds and inhibiting the formation of reactive oxygen species in cochlear cells [23,26,28]. Only one randomized clinical trial evaluating the efficacy of STS in pediatric oncology has included children with CNS tumors. ACCL0431 randomized children 1–18 years of age with multiple cancer types (total *n* = 125; *n* = 26 with medulloblastoma/CNS primitive neuroectodermal tumor) between STS and observation; patients were stratified by age, prior cranial radiation, and cisplatin infusion duration [29,30]. STS significantly decreased the proportion of patients with hearing loss (28.6% in the STS-treated group versus 56.4% in the observation group, *p* = 0.00022). For the secondary survival analysis, patients were post hoc classified as having localized (*n* = 77) versus disseminated disease (*n* = 47). No survival differences were observed for patients with localized disease. However, in the disseminated disease group, overall survival was significantly lower among patients treated with STS. While the post hoc analysis was deemed at risk of significant bias (including unmeasured confounders such as the two arms not being matched for prognostic factors) [23,30,31], the most recent clinical practice guideline provided a weak recommendation to consider STS in children and adolescents with non-metastatic cancers other than hepatoblastoma for the prevention of cisplatin-induced ototoxicity and to not routinely use STS for children with metastatic cancer [23]. STS is currently approved to be administered intravenously at a dose of 10–20 g/m^2^ over 15 min at 6h after the end of the cisplatin infusion [27]. The most commonly reported side effects include nausea and vomiting, nephrotoxicity, cytopenia, and electrolyte imbalance (hypernatremia, hypermagnesemia, hypophosphatemia, hypokalemia) [26,29,32]. Whether STS mitigates worsening ototoxicity among children with pre-existing hearing loss or among those who have developed this treatment-related complication is yet to be determined (Table 1) [23]. Additionally, future clinical trials among disease-specific populations (e.g., ACNS2031 for children with medulloblastoma) and for children with metastatic disease are urgently warranted [30].

**Table 1:** Comparison of otoprotective agents.

Agent	Indications	Chemical Class	Benefits	Adverse Effects	Dose
Amifostine	Should not be routinely used to prevent cisplatin-induced hearing loss [23]	Organic thiophosphate compound	Did not reduce hearing loss in pooled analyses [23]	Hypocalcaemia, nausea, hypotension [23]	600 mg/m^2^ IV pre-cisplatin infusion and three hours into cisplatin infusion [24]
Sodium thiosulfate	Prevention of cisplatin-induced hearing loss for children, adolescents, and young adults with non-metastatic solid tumors	Antioxidant	Significant reduction of hearing loss by approximately 50% [29,30]	Nausea, vomiting, nephrotoxicity, cytopenia, electrolyte imbalance (hypernatremia, hypermagnesemia, hypophosphatemia, hypokalemia) [26,29,32]	10–20 g/m^2^ IV over 15 min, six hours after the end of the cisplatin infusion [27]

Abbreviations: IV: intravenous.

## 3. Neurocognitive Toxicity

CNS toxicity remains a leading cause of morbidity in the treatment of pediatric brain tumors. Some of these complications occur during treatment, while others may present months or years following the completion of therapy. Surgery, radiation, chemotherapy, and novel biological or targeted treatments have all been shown to result in CNS side effects. Cognitive deficits in survivors of childhood cancer pose a substantial impact on their functional outcomes and quality of life, such as difficulties adapting to new environments, poor academic performance, social withdrawal, and, at times, challenges to living independently or completing activities of daily living.

Various aspects of therapy play a role in affecting neurocognitive outcomes in survivors of childhood cancer. The development of hydrocephalus, post-surgical complications such as cerebellar mutism, and a previous history of stroke or seizures all negatively impact cognitive function [33,34,35]. Traditional chemotherapy can have deleterious CNS effects, with added toxicity for some agents when administered intrathecally or at higher doses. Systemic therapies such as high dose methotrexate or intrathecal chemotherapy have been associated with cognitive side effects in brain tumor survivors. Side effects may include encephalopathy, myelopathy, cerebellar degeneration, ataxia, motor deficits, posterior reversible encephalopathy syndrome, seizures, and visual or hearing toxicity [36].

Radiation is commonly used in the treatment of pediatric CNS tumors. Radiation is associated with short- and long-term neurotoxicity. In the more immediate phase, pseudoprogression, worsening neurological symptoms, somnolence, and radiation necrosis are concerns, whereas long-term toxicity includes vasculopathy, neurocognitive impairment, and secondary malignancies. The dose and field of radiation impact this risk factor, with higher cumulative RT dosing leading to worse outcomes. Furthermore, patient age at the time of radiation administration plays a role [37,38,39,40,41], hence the standard radiation-sparing approach to treatment in various infant brain tumors. There is a decline in multiple neuropsychological domains due to radiation therapy, including decreased intelligence quotient (IQ), cognitive abilities, attention, concentration, processing speed, and memory. This has been shown to manifest difficulty acquiring new skills when compared to peers, rather than progressive loss of previously acquired skills. This may contribute in part to the known age-related effects on treatment, specifically radiation [42].

Several strategies have been developed to reduce the neurocognitive late effects of therapy, which include the choice of chemotherapy agents, radiation doses, and radiation techniques with reduction in exposure to surrounding healthy brain tissue, such as the use of proton radiation.

Nonpharmacological interventions have been suggested to reduce, limit, or even reverse the effect of radiation on the brain: control of hypertension and diabetes, reducing or limiting exposure to alcohol, and smoking cessation have been used in adult patients. Limiting doses to the hippocampus and supratentorial area, along with overall dose de-escalation, have also been studied (Table 2) [20,43,44].

In regard to pharmacological management, memantine has shown promise in delaying the time to cognitive decline and improving preservation of processing speed and executive function in some patents (Table 2) [45]. As a low-affinity voltage-dependent noncompetitive glutamatergic NMDA antagonist, memantine preferentially binds to NMDA receptors and prevents calcium ion influx, which in turn prevents the disruption of synaptic plasticity [45]. In early studies—when used in patients with brain metastasis, not primary CNS tumors—memantine has been shown to be associated with near-normal physiological NMDA activity despite high levels of glutamine when used as a neuroprotective mechanism. Memantine was evaluated in a phase III randomized placebo-controlled trial in patients treated with whole brain radiotherapy, where memantine treatment was initiated within 3 days of RT and continued for 24 weeks [45]. Within this study, although the primary endpoint of reduced decline in relayed recall was not reached, memantine was found to have some benefit, with a reduced probability of cognitive failure at 24 weeks (53.8% vs. 64.9%) and a longer time to cognitive decline, higher executive function at 8 and 16 weeks, and delayed recognition at 24 weeks [45]. There is limited data on the effects of memantine in patients with primary CNS tumors. Clinical trials are underway; one study includes patients older than 6 years of age receiving focal brain irradiation for a diagnosis of glioma, craniopharyngioma, ependymoma, or germ cell tumors (NCT03194906). COG is currently conducting a phase III randomized, placebo-controlled trial evaluating memantine’s effect as a neuroprotective mechanism in pediatric CNS tumor patients undergoing RT (ACCL2031).

Metformin, another oral agent, has shown promise in improving cognitive recovery in pediatric patients following radiation therapy [46]. Metformin has been shown to improve working memory by acting on neural stem cells in the subventricular zone and dentate gyrus, restoring neurogenesis following RT [46]. Early-phase randomized placebo-controlled trials demonstrated enhanced auditory-verbal recall and working memory in pediatric patients [43,44]. Metformin was overall well-tolerated in these studies, with mild gastrointestinal side effects such as diarrhea being most common [46].

Modafinil, a dopaminergic CNS stimulant commonly used for narcolepsy treatment, has shown some improvement in attention, psychomotor speed, and memory [47,48,49]. Although not all studies have shown cognitive benefit thus far [50], it has been trialed in COG’s ACCL0922 to evaluate its effect on improvement in attention, executive function, and fatigue in comparison to a placebo. Results remain pending.

Other groups have evaluated cognitive training programs in pediatric patients undergoing cranial RT. The Children’s Oncology Group (COG) ACCL10P1 is one such study that utilized a computerized cognitive training program on children 3–5 times per week.

## 4. Vascular Complications

Children treated for CNS tumors are at significant risk of cerebrovascular events (CVEs), including transient ischemic attacks, strokes (ischemic, hemorrhagic), moyamoya, and vascular malformations [51,52]. Among survivors of childhood CNS tumors, the incidence of stroke has been estimated to be as high as 267.6 per 100,000 person-years, representing a relative risk of stroke 29 times higher than in sibling controls [51]. In a population-based study of medulloblastoma survivors conducted by our group, the cumulative incidence of stroke at age 30 was 6.5% compared to 0.3% in matched controls [6]. The first stroke may occur as early as 10 years post-cancer diagnosis, with a median age of 27 years [51,52,53]. Importantly, subsequent strokes are exceedingly common; the 10-year cumulative incidence of recurrent stroke among CNS tumor survivors treated with radiation therapy has been estimated to be 31% [52]. CNS tumor survivors who had experienced a stroke had a late mortality hazard ratio that was 2.1 times higher than stroke-free survivors and were more likely to be unmarried, unemployed, disabled, and living with a caregiver [54]. Overall, CVEs convey significant morbidity and mortality for CNS tumor survivors [55,56].

The underlying mechanism of CVEs is incompletely understood but likely involves radiation-induced arteriopathy, vascular remodeling, accelerated atherosclerosis, and subsequent vessel infarction due to inflammation [57,58]. Survivors are often asymptomatic, with vascular abnormalities detected on neuroimaging screening (Figure 1) [57]. Risk factors for stroke and recurrent stroke include older age, increased time since treatment, cranial radiation therapy (dose-dependent, highest risk among patients who received ≥50 Gy), suprasellar radiation, neurofibromatosis type 1, hypertension, hypercholesterolemia, obesity, and diabetes mellitus [6,51,53,57].

Few guidelines exist for the surveillance of pediatric CNS tumor survivors at risk of neurovascular complications. For asymptomatic adult cancer survivors who received head/neck radiation therapy, the European Society of Cardiology recommends to consider carotid ultrasound imaging every 5 years, starting at 5 years after radiation and every 5–10 years thereafter [59]. The most recent survivorship guidelines from the Children’s Oncology Group advocate for the following considerations for survivors who have received brain radiation: (1) managing risk factors that predispose to CVEs (e.g., hypertension, diabetes, hyperlipidemia); (2) imaging with brain MRI and MRA as indicated; (3) revascularization interventions for moyamoya as indicated; (4) Doppler ultrasound of carotid vessels as clinically indicated (with referral to cardiology if abnormal) [60]. For survivors who received ≥40 Gy radiation to the neck, these guidelines recommend a neck doppler ultrasound 10 years post-radiation completion as a baseline [60]. However, a recent study reported that only 19.4% of CNS tumor survivors meeting screening criteria had received a carotid ultrasound, and survivors who had seen a cancer specialist in follow-up were more likely to have received appropriate screening [61]. Neuroimaging for screening asymptomatic childhood cancer survivors at risk for CVEs (modality, initiation, duration, frequency) and the use of antiplatelet therapies remain controversial [57]. There is consensus among experts that survivors with asymptomatic vascular abnormalities detected on screening imaging should have follow-up imaging every 1–2 years [57]. Future research should focus on the impact of screening for asymptomatic neurovascular disease on outcomes, the role of antiplatelet agents and statins to mitigate the risks of CVEs, and the impact of proton therapy on the incidence of CVEs [57].

## 5. Radiation Necrosis

A complication of external beam radiation in the management of pediatric CNS tumors is radiation-induced injury or radiation necrosis. It is thought to be related to direct injury to vasculature and occurs within the first two years of radiation treatment, but may be as early as 6 months following RT. In the early setting, RT necrosis is often indistinguishable from tumor progression or recurrence, which often possess challenges diagnostically [62,63,64,65].

Radiation necrosis has been postulated to be secondary to two mechanisms: vascular injury from the upregulation of pro-inflammatory markers, resulting in endothelial cell death, or the creation of a pro-inflammatory environment. Radiation necrosis may be managed with an observational approach, or, in those who are symptomatic, treatment with corticosteroids or bevacizumab is often used (Figure 1) [66]. Bevacizumab, an anti-angiogenesis agent, has been shown to decrease wound healing and increase the risk of hemorrhage and thrombosis in patients; it has also been associated with improvement in neurocognitive outcomes—specifically, memory, attention, language, and concentration—in comparison to corticosteroid therapy [63].

Compared with conventional photon radiation, proton radiation offers a theoretical advantage in sparing adjacent normal brain tissues. This may offer potential benefits with the reduction of some long-term effects of therapy such as cognitive deficits, endocrine or vascular changes, and secondary malignancies. Unfortunately, radiation necrosis remains a challenge with both modalities of RT therapy, protons and photons, with some studies showing a higher incidence and shorter time to development of radiation necrosis with proton radiation [62,65].

## 6. De-Escalation of Therapy to Reduce Toxicity

For a subset of pediatric CNS tumors with good survival outcomes, efforts are being made to de-escalate therapy to reduce toxicities while still maintaining excellent survival outcomes.

Patients with non-metastatic WNT medulloblastoma who have undergone GTR of their tumors and been treated with multimodal standard therapy have excellent progression-free survival (PFS) at greater than 90% at 5 years. Patients with WNT-driven medulloblastoma treated on the SJMB03 protocol had 100% PFS at 5 years [67]. However, the toxicities were not negligible. Of the 53 patients treated in the study with WNT-driven disease, one developed pulmonary fibrosis and died 8.3 years from diagnosis, four developed secondary malignancies, and three of these died. The current St Jude’s medulloblastoma study SJMB12 (NCT01878617) is attempting to reduce therapy for WNT-driven medulloblastoma with reduced-dose CSI as well as a reduced dose of cyclophosphamide during maintenance chemotherapy. Similarly, COG recently completed accrual to ACNS1422, which treated standard-risk WNT-driven medulloblastoma patients with reduced CSI (18 Gy) with a limited target volume boost to the tumor bed as well as a reduced chemotherapy approach (no vincristine during radiotherapy and a reduced-dose maintenance) (NCT02724579). Analysis of this study is ongoing.

Patients with CNS germinoma also have good survival outcomes but are at risk for a number of late effects from their treatment. Efforts have been made to avoid radiation, but results showed that whole-ventricular irradiation (WVI) is an essential part of treatment [68,69]. Recent international cooperative trials have demonstrated good survival outcomes with lower WVI at 24 Gy. A report from a Japanese multi-institutional study of 115 patients treated with carboplatin and etoposide followed by 24 Gy WVI showed that only one patient had tumor recurrence, with a median follow-up time of 36 months [70,71]. Early reports from the European SIOP trial also showed excellent outcomes for patients with CR treated with 24 Gy WVI [72]. Given these promising results, the current COG study (ACNS2321) is similarly examining a response-based reduction in radiation for a subset of patients with CNS germinoma (NCT06368817).

Ongoing efforts of molecular characterization and participation in large cooperative studies will help further highlight which groups of patients have the best survival outcomes and may be amenable to further therapy de-escalation. Ongoing studies such as ACNS1833 and 1831 are randomizing patients between targeted therapy and conventional chemotherapy, which may provide data on both the efficacy as well as the toxicity of each approach.

As we learn more about outcomes by tumor subtypes and treatment regimen, it will be important to use this information to de-escalate treatment when possible, thereby minimizing toxicity.

## 7. Fertility Preservation

Patients treated for pediatric CNS tumors are at an increased risk for impaired fertility. In particular, patients treated with heavy metal, alkylator, and radiation therapy are at significant risk for infertility [73]. While some of these risks are well-established, newer targeted therapies may also pose future risks to fertility, but many of these are still emerging or unknown [74]. Cranial irradiation can affect the hypothalamic pituitary axis, while alkylating chemotherapy can be gonadotoxic. Current guidelines suggest that fertility preservation measures should be discussed with patients prior to initiating chemotherapy when possible [75]. A systematic review of published reports on fertility after CNS cancer showed a polled prevalence of gonadal toxicity of 20% in survivors of CNS tumors [76]. However, the population was very heterogeneous, and the follow-up was short. Outcomes vary widely based on diagnosis and specific treatment. In a study of fertility outcomes in 62 medulloblastoma survivors, 76% of female patients and 34% of male patients had clinical or biochemical evidence of gonadal dysfunction [77].

The Children’s Oncology Group (COG) has published a report stratifying some of the most commonly used treatment protocols for CNS tumors based on fertility risk [78]. They examined the risks based on treatment agents and doses and stratified by patient sex and pubertal status. Most (7/11) CNS treatment protocols placed at least one group of patients at high risk for impaired fertility. Male patients were most commonly at a high risk for infertility, followed by pubertal females.

Based on sex and pubertal status, a number of fertility preservation options currently exist for patients. For post-pubertal males who can produce a sperm specimen, sperm can be cryopreserved. For patients who may have limited physical mobility due to the location of their CNS tumor or to surgical complications, an alternative method of collecting sperm such as electro-ejaculation may be considered [79].

For pubertal female patients, oocyte collection and cryopreservation is an established method of fertility preservation [80]. However, this requires hormone stimulation, which typically takes two weeks to achieve. For some CNS tumors, this may lead to delays in treatment that could affect survival outcome. Fertility preservation via hormone suppression with Gonadotropic Releasing Hormone agonists (GnRHa) may also be considered, but the benefit is still uncertain. A current COG study ALTE 2131 is investigating the effect of GnRHa Triptorelin on female adolescent and young adult cancer patients being treated with alkylating agents (NCT06513962).

In prepubertal female patients, ovarian tissue collection and cryopreservation with the intent of future re-implantation or in vitro growth is an option for some patients [81]. This is most often done by laproscopic oophorectomy and ideally combined with other procedures such as port-a-cath insertion [82]. In prepubertal males, testicular tissue cryopreservation is an investigational option at some institutions [83] and may be able to be combined with other procedures that the patient is undergoing to avoid delays in therapy. The efficacy of achieving live birth after cryopreservation in pre-pubertal patients is still being established. For prepubertal patients at risk for impaired fertility due to premature ovarian failure, some families may also choose close endocrinological surveillance and a discussion of possible fertility preservation methods with the patient after puberty (Figure 1).

Another important consideration in fertility preservation is the cost associated with the procedures and banking, which vary widely between and within countries. Access to certain methods can also vary based on the patient’s institution.

Patients with CNS tumors have a unique set of challenges that can affect fertility preservation. Physical and cognitive neurological deficits in patients with newly diagnosed CNS tumors may complicate discussions about fertility preservation. The discussion around fertility may also be complicated by the very poor prognosis of certain CNS tumors, such as diffuse midline glioma. For patients with cognitive changes or limited prognosis, an ethicist may be consulted to help navigate some of these decisions. With the advances of molecular diagnostics in CNS tumors, a specific treatment plan may not be decided until the results of molecular tests are obtained, sometimes 2–3 weeks after diagnosis. In these cases, the time between diagnosis and initiation of treatment may be very short, thereby limiting the time for fertility preservation. Given the many known and possible risks to fertility posed by treatment for CNS tumors, referral for oncofertility preservation is recommended when feasible.

## 8. Importance of Surveillance and Follow Up

With improved survival in children, adolescents, and young adults treated for childhood cancer, the study of chronic health conditions related to therapy remains an ongoing area of research focus. Early recognition, prevention, and initiation of treatment are essential to improve outcomes and quality of life in childhood brain tumor survivors. Monitoring the late effects of therapy is critical, as it permits the early recognition of side effects and subsequent intervention or treatment [84]. Multi-disciplinary follow-up along with access to psychosocial support is imperative.

The Children’s Oncology Group (COG)’s “Long-Term Follow-Up Guidelines for Survivors of Childhood, Adolescent and Young Adult Cancers” serve as screening and management guidelines for the late effects of therapy. Late effects are numerous and, in part, are related to the treatments that children receive—whether surgery, radiation, chemotherapy, or a combination approach—the age at which they were treated, the cumulative doses or exposure of treatments, and the location of their tumor, just to name a few. Given the multitude of effects therapy can have on patients, focusing on various organ systems after the completion of treatment is imperative. Adult survivors of childhood CNS tumors are at a high risk for long-term morbidity and late mortality related to the therapy they received, with great risk for the development of new endocrine, neurological, or sensory complications 5 years or more after diagnosis [85].

The Childhood Cancer Survivorship Study (CCSS) included 1877 CNS tumor survivors, 82% of whom suffered at least one chronic medical condition after therapy [85]. With increasing time from original cancer diagnosis, studies have demonstrated that childhood cancer survivors are less likely to receive care specific to long-term follow up [86,87]. It is at these increasing years post-cancer therapy that survivors are at the most risk for developing new medical conditions. Thus, adherence to long-term care guidelines by specialized long-term follow up clinics or dissemination of information to general practitioners and following guidelines recommendations are important.

## 9. Conclusions

Survivors of pediatric CNS tumors are highly susceptible to long-term toxicity resulting from tumor location and the treatments administered, amongst other factors. These profound late effects are associated with increased morbidity and mortality. Ongoing efforts to reduce the late effects of therapy by employing strategies to minimize toxicity, de-escalating therapy, pursuing fertility preservation, and ensuring patients undergo surveillance and follow up after the completion of therapy are imperative.

## Figures and Tables

**Table 2:** Approaches to reduce neurocognitive toxicity.

**Nonpharmacological**	**Nonpharmacological Intervention**				
	Reduce or limit CNS radiation - Limit doses to hippocampus and supratentorial area - Dose de-escalation				
	Control HTN and diabetes				
	Reduce or limit exposure to alcohol				
	Smoking cessation				
**Pharmacological**	**Drug Name**	**Mechanism of Action**	**Which Patients to Consider**	**Evidence**	**Toxicity**
	Memantine [44]	Binds to NMDA receptors and prevents calcium ion influx, which in turn prevents disruption of synaptic plasticity	Patients with CNS tumors receiving radiation therapy	Phase III RCT memantine initiated within 3 days of RT and continued for 24 weeks. Memantine found to have benefits, with reduced probability of cognitive failure and longer time to cognitive decline, higher executive function, and delayed recognition Current trials: -NCT03194906 -ACCL2031	Most common adverse effects include fatigue, alopecia, nausea, and headaches
	Metformin [42,43,45]	Improves working memory by acting on neural stem cells in subventricular zone and dentate gyrus, restoring neurogenesis following RT	Patients with CNS tumors receiving radiation therapy	Early-phase RCT demonstrated enhanced auditory-verbal recall and working memory in pediatric patients	Mild gastrointestinal side effects such as diarrhea most common
	Modafinil [46,47,48,49]	Dopaminergic CNS stimulant	Patients with CNS tumors receiving radiation therapy	Improvement in attention, psychomotor speed, and memory. Current trials: ACCL0922	Most commonly insomnia, headaches, nausea, and anxiety

Abbreviations: NMDA: N-methyl-D-aspartate; RCT: randomized control trial; RT: radiation therapy.
